# Defining TestOps: Collaborative Behaviors and Technology-Driven Workflows Seen as Enablers of Effective Software Testing in DevOps

**DOI:** 10.1007/978-3-030-58858-8_26

**Published:** 2020-08-18

**Authors:** Michal Doležel

**Affiliations:** 6 IT University of Copenhagen, Copenhagen, Denmark; 7 University of British Columbia, Vancouver, BC Canada; University of Economics, Prague, 130 67 Prague, Czech Republic

**Keywords:** Continuous testing, Testing in production, Shift-left testing, Shift-right testing, Software testers, Software-testing skills, Collaboration

## Abstract

**Context:** DevOps is an increasingly popular approach to software development and software operations. Being understood as mutually integrated, both activities have been re-united under one single label. In contrast to traditional software development activities, DevOps promotes numerous fundamental changes, and the area of software testing is not an exception. Yet, the exact appearance of software testing within DevOps is poorly understood, so is the notion of TestOps. **Objective:** This paper explores TestOps as a concept rooted in industrial practice. **Method:** To provide a pluralist outline of practitioners’ views on *What is TestOps*, the YouTube platform was searched for digital content containing either “TestOps” or “DevTestOps” in the content title. Through a qualitative lens, the resulting set was systematically annotated and thematically analyzed in an inductive manner. **Results:** Referring to DevOps, practitioners use the notion of TestOps when characterizing a conceptual shift that occurs within the area of software testing. As a matter of fact, two dominant categories were found in the data: (i) TestOps as a new organizational philosophy; (ii) TestOps as an innovative software technique (i.e. process supported by technology). A set of high-level themes within each of these categories was identified and described. **Conclusion:** The study outlines an inconsistency in practitioner perspectives on the nature of TestOps. To decrease the identified conceptual ambiguity, the proposed model posits two complementary meanings of TestOps.

## Introduction

During the past years, the concept of DevOps (Development-Operations) has firmly established itself as an important landmark in the software domain. DevOps can be defined in a number of ways, including a “set of practices”, a “development methodology”, a “cultural movement”, an “organizational approach”, and “infrastructure” [1]. Hence, little clarity around DevOps exists, and many see DevOps representing more than one of these aspects. Bringing a sort of consensus and partly clearing the “buzz” [2], four DevOps dimensions were proposed to describe DevOps as a multifaceted phenomenon. Specifically, ***C****ulture*, ***A****utomation*, ***M****easurement* and ***S****haring* (CAMS) have been viewed as the key focus areas when adopting DevOps [3, 4].

Regarding *Sharing,* i.e. one of the organizational vehicles crucial for DevOps, practitioner literature claims that team members should be encouraged to share knowledge and practices to make DevOps successful [1]. In essence, DevOps teams are considered autonomous, and exhibit more blurred responsibilities of software professionals than in traditional teams [5]. For example, previously independent functions, such as independent software testers, may become embedded in DevOps teams in an attempt to strengthen the teams’ autonomy, self-sufficiency and cross-disciplinarity [6].

Not only due to the *Sharing* element in DevOps, software testing is an area which is expected to become significantly influenced by the adoption of DevOps principles (cf. [7]). In fact, new test approaches and strategies are needed for DevOps, along with the new skills of test personnel [8]. The emergence of *continuous testing* is one particular facet of this shift, being enthusiastically promoted as “the key for DevOps” [9]. Yet, from an overall perspective, the possible synergy between software testing and DevOps is still poorly understood, having been discussed in only a handful of empirical studies [10] and practitioner reports [6, 9] so far. Also, in practitioner forums, the discussion seems to be somewhat fragmented. Two salient topics related to DevOps have emerged – the notion of *shifting left,* and the notion of *testing in production*. Simply put, the former is an idea of involving testers early in the software development life cycle [11]; the latter idea proposes to start “using real users and real environments to find bugs that matter” [12].

In addition to the previous ideas, some practitioners recently coined the term *TestOps* [12, 13]. Nevertheless, this broad notion seems to add even more confusion on the top of the already ambiguous DevOps concept. Presently, there is little clarity about what the term TestOps means to practitioners today, or could mean in the future. This gap in knowledge has motivated the present exploratory qualitative study. Using an inductive approach, a thematic analysis of available YouTube content related to TestOps was carried out. As a result, a conceptual map was constructed, rendering an inconsistency in practitioner perspectives on the nature of TestOps. By exploring the influential, yet in Software Engineering (SE) research an under-used digital media [14], the study reports on the significance of TestOps, as being promoted by early adopters in the field. The paper contributes to the emerging line of research on software testing in DevOps.

The paper is organized as follows. Following Introduction, the research approach is described in Sect. 2. Section 3 presents a summary of findings. Section 4 concludes the paper by discussing the principal takeaways and limitations of the study.

## Research Approach

The practice-driven research described in this paper took part within a broader collaborative initiative between academia and a global antivirus company. Our common focus has been on increasing the understanding of the changing role of software testers nowadays (see also [8]). The main aim of this small-scale study was to propose a provisional definition of TestOps/DevTestOps by analyzing how practitioners across the world characterize this emerging concept. The study was motivated by a lack of clarity in how the concept fits into a broader picture of DevOps and continuous practices studied earlier [2–4]. The research question posed here therefore is: *How do early*-*adopters define and promote TestOps/DevTestOps as a concept?*

### YouTube as Data Source.

We used YouTube – a content-sharing media platform – as the main source of empirical evidence. While software practitioners follow a plethora of knowledge sources to learn about new technologies and influential ideas, media platforms like YouTube seem to be particularly popular among them [14]. In fact, these platforms represent an inexpensive, easily accessible option for staying updated about the latest trends in the field. Presently, only a small portion of SE researchers use this type of research data [14]. Arguably, this is because these platforms are not viewed by the academic part of the SE community as a credible-enough source of information. Yet, Garousi, Felderer, and Mäntylä [15] explicitly categorize audio-video media within the same category as blogs, both exhibiting “moderate outlet control and credibility”. So far, only the merit of the latter data source has been clearly articulated by SE researchers [16]. Fortunately, regarding the use of YouTube, inspiration can be taken from other disciplines, e.g. from medicine. In this field, YouTube is considered a legitimate source of research data, for example when exploring specific topics with limited existing knowledge [17], or when mapping emerging sources of information available to patients [18].

In our view, publicly available video platforms offer a vivid, pluralist view on coming SE trends rooted in practice. Having said that, an important methodological aspect should be taken into consideration. That is, the content available at these platforms may be significantly shaped by marketing activities of software companies (e.g. tool-producers). Similarly, additional individual actors may cause that the obtained perspective will fundamentally differ from a mainstream one, i.e. from a hypothetical view of an “average” software practitioner. To label these actors with influence, we propose the term *brand evangelists* borrowed from marketing research. The term denotes individuals who act on their own with the intention to persuade others to adopt a product or idea [19]. Typically, brand evangelists do so via social media platforms.

Regarding the material included in this study, the majority of videos was recorded at practitioner conferences focused on software testing, or during software testing webinars. In SE, both the above mentioned platforms represent crucial vehicles for knowledge dissemination among practitioners [20]. In this study, we did not attempt to identify brand evangelists nor categorize the analyzed content by involving any similar criteria. In general, we hold that it is presently unclear how practitioners judge the credibility of conference speakers in terms of sensing them as possibly *selling* or *evangelizing*. Indeed, many practitioners consider conference speakers to be highly influential figures and *de*-*facto* thought leaders of industrial practice [21].

### Data Collection.

Using two keywords (“TestOps”, “TestDevOps”), a search for relevant content at YouTube was performed. The search results were sorted according to relevance, which is the default setting. All content displayed in the main result section and containing “TestOps” or “DevTestOps” [and variants such as Dev(Test)Ops] in the title was considered. The results listed in the sections “Related to your search” and “For you” were not included. After an initial screening, further excluded was: (i) trade content dealing solely with a technological aspect of commercially available solutions; (ii) all content in different languages than English; (iii) post-conference interviews with the presenters. Additionally, the most recent presentation from the same author presenting the topic at more than one venue was selected. Whenever possible, the decision was based on the examination of the presentation details (e.g. the first slide), not YouTube metatags of the videos.

### Possible Replicability Issues.

With regard to the nature of data, full replicability cannot be guaranteed, as researchers have generally little control over media ranking algorithms embedded in the platforms like YouTube [15]. In the present study, this was partly mitigated by using a ‘clean’ browser, carrying out the search with the same keywords multiple times, and focusing only on the non-personalized search results. Also, the consistency of the results was checked using a different computer homed in a different network infrastructure. The final list of included content is available in on-line Appendix (https://cutt.ly/ciQgedC).

### Data Analysis.

The principles of inductive thematic analysis [22, 23] were followed to analyze the material, which comprised of 8 h and 19 min of recordings. To get familiar with the data, we firstly played every recording in full-length. During this process, memoing [22] was used to capture preliminary ideas about the data. This phase was followed by a systematic annotation (open-coding) of the content using the video analysis feature in our qualitative data analysis software (MAXQDA Plus 2020, r. 20.0.7). The descriptive codes derived from individual recordings were than cross-compared and re-organized into larger categories. The resulting conceptual map was constructed in an inductive manner through constant comparison [22].

## Results: What Is TestOps?

**Fig. 1.**
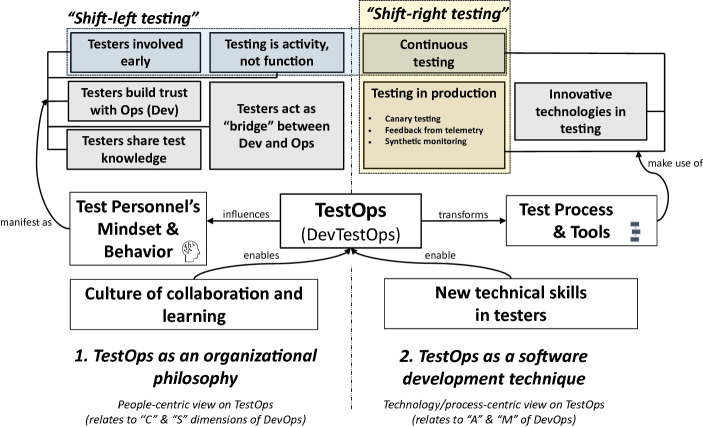
Conceptual map of TestOps

Based on our analysis, a level of disagreement among practitioners regarding the meaning of the term TestOps was identified. While concise definitions of the term were rarely given in the analyzed talks, it was still possible to derive two broad super-categories of meanings that practitioners attribute to TestOps (Fig. 1). These are as follows.

### TestOps as a Pattern of Collaborative Behavior Associated with a Shift in the Test Personnel’s Mindset and Organization.

This super-category covers the human and organizational aspects of TestOps described by the following high-level themes. To begin with, the theme *Testers [need to be] involved early* was central to the discussion. While this is not a new claim, it is in contrast with the everyday reality of sequentially-managed software projects, in which independent testers dominate a separate testing phase near the end of the software development lifecycle. Inversely, TestOps-inspired testers were encouraged to leave silo-ed test centers and move back to development teams *(Testing is activity, not [organization] function)*. Highlighting the aspects of collaboration, testers were further encouraged to *Act as “bridge” [or “glue”] between Dev and Ops* (hence the term Dev***Test***Ops). Also, they were asked to offer their testing-related expertise to both developers and operations specialists, and to guide these professionals through software testing problems *(Testers share test[ing*-*related] knowledge)*. Proactive trust and relationship building *(Testers build trust with Ops/Dev)* initiated by testers was promoted as a mean for lowering existing organizational barriers (e.g. issues related to organizational structures). The practitioners also held that the described patterns of behavior did not happen automatically. Therefore, creating a *Culture of collaboration and learning*, resulting in the “right mindset” of people, was suggested as a key enabler.

### TestOps as a Technology-Intensive Set of Software Practices.

In contrast to the previous view, this super-category covers the technology-related aspects of TestOps summarized as the following high-level themes. First and foremost, the practitioners argued that software testing in TestOps needed to be conducted in a rapid and holistic way. Again, this new form overarches the traditional (phase-based) understanding of software testing. In that sense, *Continuous testing* was promoted as a key practice that was to introduce automated tests as an integral part of deployment pipelines (i.e. automated processes driving the integration, building and deployment of software versions). As the professionals argued, while testers did not need to become DevOps engineers, they needed to acquire skills allowing them to support DevOps engineers and/or developers in their effort. In addition, another impactful shift was discussed: The scope of software testing was claimed as newly ranging beyond test (or non-production) environments. To label this new phenomenon, the term *Testing in production* (TiP) was introduced. The TiP theme was conceptually rich. Interestingly, some practitioners explicitly cited the work of Seth Eliot [12] as a source of the ideas presented by them. Therefore, Eliot’s categorization informed also our analysis.

Eliot’s “TestOps model of software testing” [12] promotes three TiP-related activities discussed by the practitioners to a varying extent: 1) evaluating the impact of new features provided to a small proportion of end-users (canary testing); 2) using telemetry data from production to design better tests; 3) using “active monitors” or “synthetic monitoring” at production environments. All these activities were suggested as a source of rich production data that could be used by testers for improving test coverage and the depth of their testing. Differently put, by increasing testers’ understanding of end-user interactions with the application, testers could do their job better.

Finally, varying significantly in depth and focus, some practitioners discussed the impact of new technologies on TestOps (e.g. cloud, big data, and artificial intelligence being the technologies that caused “digital disruption” in many segments) (*Innovative technologies in testing*). Details regarding this theme are considered beyond the scope of this paper. Taking into consideration all the above technology-related aspects, the key enabler was conceptualized as *New technical skills in testers.*

While not explicitly defined in the analyzed video sample, the notions of *Shift*-*left* and *Shift*-*right testing* are conceptually related to TestOps. The proposed understanding is portrayed in the conceptual map as well, and further discussed in Sect. 4.

## Discussion and Conclusion

Exploring the views of TestOps advocates, this paper brings initial insights into the nature of TestOps. The presented analysis demonstrates that according to the practitioners, the notion comes with a set of powerful ideas. When embraced by industry, the ideas may cause a shift in the field of software testing, which was previously dominated by manual testing [8]. Using a conceptual map, two meanings of TestOps were explored. First, a people-centric view was conceptualized as the cornerstone of TestOps. This perspective binds TestOps with the ***C****ulture* and ***S****haring* dimensions in DevOps. In contrast to this view, it was proposed that some practitioners used the term “TestOps” to label a different area of interest: the emerging field of software technology concerned with new tools, workflows and processes for effective test automation supporting DevOps teams. The latter meaning of TestOps goes back to the ***A****utomation* and ***M****easurement* DevOps dimensions. This is to highlight that a potential for bringing significant technological innovations to the SE field exists by establishing synergies between test tools and other tools used by either developers or operations.

As portrayed in the conceptual map, the term *Shift*-*left testing* transcends the boundary between the two perspectives, i.e. refers both to people and technology. This is due to the term being a conceptual umbrella for both an organizational approach (*Testers involved early [and continually]*) and a related technology-driven practice (*Continuous testing*). By contrast, the term *Shift*-*right testing* refers to the aspects connected with two technology-related concepts – *Continuous testing* and *Testing in production*. Interestingly, while the term *Shift*-*left testing* originated from a 2001 vision [11], the term *Shift*-*right testing* appears to be of a more recent origin. The suggested metaphor of “right” vs. “left” becomes obvious when one considers a V-shaped lifecycle model, such as the one proposed by Rook in the late 1980s [24]. In Rook’s model, the logical beginning (i.e. requirement specification) of SE activities was located on the left side, and the logical end (i.e. software operation and maintenance) on the right side. Therefore, shift-left means *earlier* than during the separate testing phase following coding. In contrast, shift-right means *later* than during that phase, i.e. in production.

Regarding the two high-level perspectives of TestOps (i.e. people vs technology), this paper does not suggest either of them as dominating. Quite to the contrary, SE practice can possibly take the best from the TestOps-related ideas by combining both views [25]. However, as our data show, not all the TestOps promoters followed this route. Not only that – some of the presentations seemingly made use of the term TestOps (DevTestOps) only as of a catchy title to attract the attention of conference audience (see Sect. 2). Likewise, it is important to mention that TestOps overlaps with already existing concepts such as continuous testing [4] and testing in production [9, 12]. As made clear in one of the analyzed videos by Lisa Crispin, a software testing thought leader, one of the reasons for using the term TestOps/DevTestOps is: “People see the word DevOps … [and they may think there is] no tester in DevOps”. Accordingly, the terms TestOps and DevTestOps were coined to highlight the importance of software testing in the DevOps world. Differently put, software testing, as a domain of expertise, is not to cease to exist. Yet, testing activities may become cross-fertilized into the total of development and operational activities performed by autonomous DevOps teams [26].

Methodologically speaking, this study has important limitations. First and foremost, exploring grey literature relates to a specific set of challenges [15] outlined in Sect. 2. Regarding the scope of this study, the YouTube content was considered for our analysis only when explicitly containing the word “TestOps” or “DevTestOps” in the title. This approach to acquiring empirical data was chosen to mimic the behavior of practitioners performing an initial mapping of a new phenomenon by searching through available YouTube videos. However, the chosen approach obviously did not cover the complete landscape of software testing in DevOps, as only a fraction of available knowledge was mapped. In our subsequent research, we plan to conduct a broader, multi-vocal study focused on getting a more comprehensive perspective on the state of software testing in DevOps.
